# “These Aren’t the Strains You’re Looking for”: Recovery Bias of Common *Campylobacter jejuni* Subtypes in Mixed Cultures

**DOI:** 10.3389/fmicb.2020.00541

**Published:** 2020-04-09

**Authors:** Benjamin M. Hetman, Steven K. Mutschall, Catherine D. Carrillo, James E. Thomas, Victor P. J. Gannon, G. Douglas Inglis, Eduardo N. Taboada

**Affiliations:** ^1^Department of Biological Sciences, University of Lethbridge, Lethbridge, AB, Canada; ^2^National Microbiology Laboratory at Lethbridge, Public Health Agency of Canada, Lethbridge, AB, Canada; ^3^National Centre for Animal Diseases, Canadian Food Inspection Agency, Lethbridge, AB, Canada; ^4^Canadian Food Inspection Agency, Ottawa Laboratory (Carling), Ottawa, ON, Canada; ^5^Lethbridge Research and Development Centre, Agriculture and Agri-Food Canada, Lethbridge, AB, Canada; ^6^National Microbiology Laboratory, Public Health Agency of Canada, Winnipeg, MB, Canada

**Keywords:** *Campylobacter jejuni*, microbiological surveillance, culture methods, enrichment, molecular subtyping

## Abstract

Microbiological surveillance of the food chain plays a critical role in improving our understanding of the distribution and circulation of food-borne pathogens along the farm to fork continuum toward the development of interventions to reduce the burden of illness. The application of molecular subtyping to bacterial isolates collected through surveillance has led to the identification of strains posing the greatest risk to public health. Past evidence suggests that enrichment methods for *Campylobacter jejuni*, a leading bacterial foodborne pathogen worldwide, may lead to the differential recovery of subtypes, obscuring our ability to infer the composition of a mixed-strain sample and potentially biasing prevalence estimates in surveillance data. To assess the extent of potential selection bias resulting from enrichment-based isolation methods, we compared enrichment and non-enrichment isolation of mixed subtype cultures of *C. jejuni*, followed by subtype-specific enumeration using both colony plate-counts and digital droplet PCR. Results differed from the null hypothesis that similar proportions of *C. jejuni* subtypes are recovered from both methods. Our results also indicated a significant effect of subtype prevalence on isolation frequency post-recovery, with the recovery of more common subtypes being consistently favored. This bias was exacerbated when an enrichment step was included in the isolation procedure. Taken together, our results emphasize the importance of selecting multiple colonies per sample, and where possible, the use of both enrichment and non-enrichment isolation procedures to maximize the likelihood of recovering multiple subtypes present in a sample. Moreover, the effects of subtype-specific recovery bias should be considered in the interpretation of strain prevalence data toward improved risk assessment from microbiological surveillance data.

## Introduction

Campylobacteriosis is among the leading bacterial foodborne infections worldwide, commonly manifesting as an inflammatory disease of the intestine, and sometimes results in bloody diarrheal syndrome, or more serious auto-immune sequelae (Acheson and Allos, 2001). The causative agents include several species from the genus *Campylobacter*, with the majority of infections attributed to *C. jejuni*. When adjusted for significant under-reporting, the per capita rate of campylobacteriosis cases in Canada was recently estimated to be 447 cases per 100,000 (Thomas et al., 2013), and it is among the most prevalent bacterial foodborne illnesses worldwide (Kaakoush et al., 2015). The epidemiology of *C. jejuni* is complex, with the majority of cases of human illness thought to be sporadic (Silva et al., 2011). Although it is generally accepted that consumption and handling of contaminated poultry products is the primary source of exposure leading to human infection, *C. jejuni* is found in a wide range of animal and environmental reservoirs (Williams and Oyarzabal, 2012; Whiley et al., 2013), providing additional routes for the introduction of *C. jejuni* into the food chain as well as non-food-related pathways of exposure (Pintar et al., 2016). Molecular subtyping of *C. jejuni* isolates recovered from samples throughout the food chain and the environment has helped to shed light on routes of transmission from various reservoirs to the human population, while providing important insights on the distribution and prevalence of *C. jejuni* subtypes that pose an increased risk to human health (Wilson et al., 2008; Müllner et al., 2009; Sheppard et al., 2009).

A significant challenge to any microbial surveillance program is ensuring that isolates obtained through sampling of the targeted reservoirs are representative of the population in circulation. To date, several studies have noted the potential for biased recovery in the isolation of foodborne pathogens such as *Listeria monocytogenes*, *Salmonella*, and *Campylobacter* (Gorski, 2012; Williams et al., 2012; Zilelidou et al., 2016). The use of different isolation protocols and media has been shown to influence the frequency of detection and can affect the diversity of *C. jejuni* subtypes observed in mixed-strain populations (Williams et al., 2012; Ugarte-Ruiz et al., 2013). A recovery step in enrichment broth was originally suggested for cases where low numbers of cells may be present (Hutchinson and Bolton, 1984), where cells may be under physiological stress (Bovill and Mackey, 1997), or to help resuscitate cells in the viable but non-culturable state (Jones et al., 1991). Subsequent culturing using antimicrobial supplements and incubation under microaerobic conditions is then used to enhance recovery of *Campylobacter* cells when competing background microflora may be present in the sample. Currently, enrichment media are widely adopted in *Campylobacter* isolation protocols from matrices such as meat, animal feces, and water, including standard methods recommended by the International Standard Organization (International Organization for Standardization, 2006) and the U.S. Food and Drug Administration (Hunt et al., 1998).

Several studies have compared non-enrichment and enrichment-based recovery methods and found that sample pre-enrichment could improve *Campylobacter* recovery over non-enrichment methods (Habib et al., 2011), as has been suggested for isolation from cattle feces (Garcia et al., 1985; Atabay et al., 1998; Stanley et al., 1998; Gharst et al., 2006). However, there is significant evidence suggesting that isolation methods employing a pre-enrichment step may favor certain subtypes and thus bias the subtype representation obtained (Devane et al., 2013; Ugarte-Ruiz et al., 2013). It is not known, however, to what extent different isolation methods affect the distribution of subtypes recovered from animal or environmental samples. If some subtypes are preferentially suited to growth under particular isolation conditions, this may affect downstream assessment of their relative prevalence. This outcome would obscure our understanding of the population circulating in various reservoirs and distort the assessment of their epidemiological significance and, ultimately, the public health risk that they present.

The objective of the current study was to examine the effects of culture conditions on the recovery of *C. jejuni* subtypes from multi-strain (i.e. mixed) samples. A significant challenge in assessing the effect of culture methods on the recovery of subtypes from naturally contaminated matrices is the inherent uncertainty in the composition and proportions of subtypes present in the original sample. To mitigate this uncertainty, we developed a controlled recovery experiment using normalized mixtures comprising defined *C. jejuni* subtypes in broth culture. Cells in these mixtures were re-isolated using enrichment and non-enrichment culture methods and the relative recoveries of each subtype were then measured by subtype-specific enumeration. This experimental set-up was used to test the hypothesis that enrichment-based culture methods differentially affect recovery in a subtype-dependent manner. It was also used to test the hypothesis that enrichment conditions preferentially select for *C. jejuni* strains representing common subtypes that are historically prevalent in Canadian surveillance data. Finally, to address uncertainty in the relative abundance of cells from each subtype at the start and throughout the progression of the experiment, we performed additional parallel overnight recovery on one of the strain mixtures and measured the relative amounts of DNA from each subtype in the total mixture using digital-droplet PCR.

## Materials and Methods

### Identification of *Campylobacter* Subtypes for Recovery Experiments

Subtypes from the Canadian *Campylobacter* Comparative Genomic Fingerprinting database (C3GFdb) were ranked by frequency and assessed for sources of isolation. Data for non-human sample sources were aggregated into six major groups consisting of: “Cattle,” “Poultry,” “Environmental,” “Other Bird,” “Other Animal,” and “Other Food” in order to compare the source distributions of the ten most common subtypes from each database. *Campylobacter* isolate data from the C3GFdb was examined to identify three “common” or prevalent subtypes; an additional set of nine “uncommon” or rare subtypes was also identified. Three multi-strain mixtures comprising four strains from distinct CGF subtypes were designed for controlled recovery experiments, which allowed for subtype-specific enumeration. Each mixture was chosen to be representative of a variety of sampling sources (Table 1) and designed to contain one common and three uncommon subtypes in order to test the hypothesis that subtypes exhibit different rates of recovery. Relative subtype prevalence was confirmed using data from the international *Campylobacter* pubMLST database (Jolley and Maiden, 2010) where possible.

**TABLE 1:** Strains of *C. jejuni* used in this study and colony counts from repeated microbiological recovery experiments.

Mixture^†^	Strain	Source^‡^	CGF Subtype	C3GFdb frequency and rank^¥^	MLST^§^	Non-enrichment Recovery		Enrichment Recovery	
							
						Trial 1 (*n* = 100)	Trial 2 (*n* = 88)	Trial 1 (*n* = 100)	Trial 2 (*n* = 83)
A	CI_5178	W	957.1.1	274 (1)	ST-45	42	52	90	73
A	CI_4685	W	844.3.1	13 (2)	ST-692	12	11	6	10
A	CI_5043	W	540.1.3	3 (3)	ST-1224	31	18	2	0
A	CI_5039	W	540.1.4	1 (4)	ST-4029	15	7	2	0

						**Trial 1 (*n* = 99)**	**Trial 2 (*n* = 100)**	**Trial 1 (*n* = 99)**	**Trial 2 (*n* = 100)**

B	CE_M_10_4053	P	169.1.2	593 (1)	ST-982	32	26	45	49
B	CI_2669	W	782.4.2	27 (2)	ST-2524	20	28	16	17
B	07_2680	H	83.7.1	38 (3)	ST-918	18	16	8	5
B	CGY_HR_241	H	27.1.3	24 (4)	ST-452	29	30	30	29

						**Trial 1 (*n* = 98)**	**Trial 2 (*n* = 98)**	**Trial 1 (*n* = 99)**	**Trial 2 (*n* = 99)**

C	07_1875	H	735.5.1	217 (1)	ST-42	34	18	52	53
C	CI_4820	W	812.2.1	51 (2)	ST-42	23	46	21	29
C	CE_R_11_0073	P	18.1.2	71 (3)	ST-1698	13	12	0	1
C	CE_R_11_0249	P	123.1.2	67 (4)	ST-51	28	22	26	16

*^†^Mixture indicates the pooled strain mixtures used in recovery experiments. ^‡^Source: W – Water; P – Poultry; H – Human. ^¥^Subtype frequency indicates the number of isolates of matching subtype in the Canadian *Campylobacter* Comparative Genomic Fingerprinting database (C3GFdb). Rank number in parentheses indicates rank given to each subtype within its mixture, corresponding to the respective frequency of its subtype cluster in the C3GFdb. ^§^ MLST Sequence Type.*

### Preparation of Pooled Spike-in Mixtures for Recovery Experiments

Pure cultures of the 12 selected *C. jejuni* strains were revived from frozen glycerol stocks stored at −80°C by sub-culturing a 20 μl loop of frozen culture into brain heart infusion broth (BHIB; Fisher Oxoid CM1135), and incubating for 24 h in a tri-gas (85% N_2_, 10% CO_2_, 5% O_2_) microaerobic atmosphere (MAA) incubator at 42°C. A 100 μl aliquot was then spread onto blood agar (BD 211037, BBL infusion agar base supplemented with 7% sheep blood) and grown overnight in MAA for subsequent culture harvesting and DNA extraction for CGF analysis to verify the subtype as described below. Following CGF verification, a 20 μl loop of the remaining culture was harvested and re-cultured into BHIB for 24 h and growth was assessed spectrophotometrically by measuring the optical density at 600 nm (OD_600_). Cultures selected for each sample mixture were normalized to a consistent optical density using sterile BHIB, and 1 ml volumes from each of the normalized broth cultures were co-inoculated into 10 ml of phosphate-buffered saline (1 × PBS, pH 7.5) to create the multi-strain spike-in mixture. This procedure was repeated in duplicate for each of the three strain mixtures. Pooled samples were then vortexed, and ten-fold serial dilutions from 10^–1^ to 10^–5^ were prepared in BHIB.

### Recovery of Isolates From Spike-in Experiments

#### Non-enrichment Method

For each of the three pooled sample mixtures, 100 μl from each dilution was spread onto five petri dishes containing Charcoal-Cefoperazone Deoxycholate Agar (CCDA) (Fisher Oxoid CM0739) without antibiotic supplement, and incubated in MAA at 42°C. After 24–48 h, dishes were assessed for suitable growth (e.g. containing approximately 20 to 50 well-formed, separate colonies per dish) and the dilution series that best matched suitable growth was selected for subsequent steps. Culture dishes were shuffled into random order, and a total of 100 colonies were arbitrarily chosen by selecting every isolate from the first and subsequent dishes until a total of 100 colonies were selected. Each of these colonies were then individually streaked to a petri dish containing CCDA. Following 24–48 h of incubation on CCDA in MAA at 42°C, cells originating from single isolated colonies were sub-cultured onto BBL blood agar and grown in MAA at 42°C for 24–48 h for subsequent DNA extraction.

#### Enrichment Method

To test the effects of including an enrichment step on subtype recovery from the pooled sample mixtures, 100 μl from each dilution of the spike-in mixture was first transferred to 20 ml of Bolton Broth (BB) (Fisher Oxoid CM983) with modified BB supplement (Fisher Oxoid SR0208E) and incubated for 24 h in a MAA at 42°C. Following enrichment, 100 μl of liquid BB culture was then processed in the same manner as described above.

### DNA Extraction and Subtype Verification by PCR

Cell biomass was harvested from BBL blood agar using a 20 μl loop and genomic DNA was extracted using a modified protocol of the Epicenter Masterpure DNA Extraction kit (Epicenter MC85200). Briefly, biomass was suspended into 300 μl of Cell and Tissue Lysis Solution (MTC096H) containing RNAaseA (1 μl, 5 mg/ml) and proteinase K (5 μl, 50 mg/ml) and heated at 65°C for up to 60 min to allow lysates to clear. Samples were then cooled on ice and 175 μl of chilled MPC protein precipitation solution (MMP095H) was added to each sample, followed by vortexing and centrifugation to remove cellular debris. Ethanol precipitation was used to recover the DNA from the resulting supernatant. The DNA pellets were suspended in buffer containing 1 × Tris-EDTA (pH 8.0) and stored at −20°C until verification by Comparative Genomic Fingerprinting (CGF) (Taboada et al., 2012). To determine the frequency of recovery for each of the four subtypes an abbreviated version of the 40-loci CGF assay, based on a subset of two of the eight five-plex PCRs required for the assay, was used to partially subtype each isolate to facilitate subtype-specific enumeration.

### Investigating Growth Dynamics in Mixed Culture Using Digital Droplet PCR (ddPCR)

#### Primer Design

The growth dynamics of mixture “C”, containing *C. jejuni* strains 07_1875, CI_4820, CE_R_11_0073, and CE_R_11_0249 were investigated using ddPCR. Diagnostic loci for each *C. jejuni* strain were identified by comparison of their CGF subtypes and identifying loci unique to each subtype within the four-strain mixture. Primers and probes were then designed for these sequences using the online version of Primer3^1^. The sequence of a single-copy conserved core gene (*cj0102*) was identified from draft whole-genome sequence analysis (unpublished work) and used as a control in the ddPCR experiment. Primer and probe sequences for each marker are listed in Table 2.

**TABLE 2:** Sequences and modifications of primers and probes designed for digital droplet PCR assay.

Strain	Target	Product	Sequence	Modification
CE_R_11_0249	cj0033-F	Primer	TGGGATAAAAGGGGTGAGAA	
	cj0033-R	Primer	CGTGAAGCCAAGTAAAACCAA	
	cj0033-Hyb	Probe	TGTTTCGAGAATTCGGGATTTTATGG	FAM
All (Control)	Cj0102-F	Primer	CAAAGCACAAAAAGTGAGATTT	
	Cj0102-R	Primer	CAACATTGTGAATAAGCTCCAT	
	Cj0102-Hyb	Probe	TGCTCCTTATGCAAAAGGTGGT	HEX
CI_4820	cj0569-F	Primer	TTGGTTTGGACATTTAGCATC	
	cj0569-R	Primer	GCTAGTGTTTGTCTATGTTGTC	
	cj0569-Hyb	Probe	TGATTGGTGTGGATCTAGTGGAGG	FAM
CE_R_11_0073	cj1431c-F	Primer	AATTGCAGGAAGGGATGATG	
	cj1431c-R	Primer	CAAATTTGCCCAAGGAATCA	
	cj1431c-Hyb	Probe	TGGTTTTAAATTCGGTTTTGTATGGAGA	FAM
07_1875	cj1550c-F	Primer	GGAAAGATGGTTGAATGGAAAG	
	cj1550c-R	Primer	TCTAAGGCTAACAAAGCATCG	
	cj1550c-Hyb	Probe	AGCAAGTAATGTGAATATGCCTAGCGT	FAM

#### Growth Dynamics Experiments

Pure broth cultures for each strain were prepared and normalized using the same methods as described above. Ten milliliters from each normalized culture were then combined and vortexed to create the spike-in inoculum, and 1 ml from the mixture was added to each of 18 tubes containing 25 ml BB (i.e. “enrichment) and 18 tubes containing 25 ml BHI (i.e. “non-enrichment”). Tubes were then vortexed and incubated in MAA at 42°C. At 4-h intervals, three tubes of each BB and BHI were removed for ddPCR analysis. Tubes were centrifuged at 9000RPM for 10 min and after discarding of the supernatant, DNA was extracted from the remaining cell pellet as described above.

#### Quantification by ddPCR

Genomic DNA from each of the samples in the overnight trial was quantified using the Quant-iT broad-range DNA quantification kit (Thermo Fisher Scientific Q33130) following manufacturer’s specifications. The DNA was then diluted to 10 ng/ul in water and digested using NciI for approximately 120 min at 37°C. Digested DNA was then diluted to 0.002 ng/ul for use in the generation of droplets following the manufacturer’s recommendations (Bio-Rad Qx100), then used in a 40-cycle PCR with the following settings: pre-denature (95°C) 10 min; denature (94°C) 30 s; annealing (60°C) 30 s; extension (60°C) 1 min; inactivation (98°C) 10 min. The final PCR product was then loaded and read using the ddPCR droplet reader (Bio-Rad QX100).

### Statistical Analyses

Statistical analyses were performed using the R language for statistical computing (version 3.3.1) (R Core Team, 2016). Unless otherwise indicated, all tests were performed using a Type I error rate of α = 0.05. Figures were generated from results using the R package ggplot2 (Wickham and Winston, 2015).

#### Analysis of Effect of Isolation Method

The Chi-square goodness of fit test (“chisq.test”) was used to assess the hypothesis that isolates from each subtype would be recovered in equal proportions under both enrichment and non-enrichment isolation methods. Analysis of variance (“aov”) was performed to test the null hypothesis that isolation protocol had no effect on the distribution of recovered isolates from each mixture across each of two independent trials. The dependent variable assessed was the recovered frequency of each subtype, and the effects of “subtype” and “isolation method” were assessed as independent variables.

#### Analysis of Effect of Cluster Size

To assess the relationship of historic subtype prevalence (i.e. cluster size) on the frequency of subtypes recovered post-isolation, the strains within each mixture were assigned a descending rank based on the size of the CGF subtype cluster from which they were derived (e.g. ranked one to four, with “Rank 1” corresponding to the CGF cluster containing the greatest number of isolates) (Table 1). Analysis of variance was then used to assess the difference between the frequency of isolates recovered from each rank, and whether there was a significant effect of isolation method on this recovery.

#### Comparison of Mean Cell Counts From ddPCR

Copy number results from the ddPCR analysis for each diagnostic marker were compared against those from the control marker (cj0102) to establish relative amounts of DNA in the total mixture corresponding to each strain. These ratios were then corrected so that each strain accounted for 25% of the total mixture at initiation of the recovery experiment (time = 0h). For each subsequent measurement, the ratio of marker to control was divided by the total abundance of the four strains in the mixture to establish the amount of DNA relative to each strain in the mixture at each time point. These results were then averaged across the triplicate trials, and the mean relative abundance for each strain was compared between enrichment and non-enrichment recovery methods at each time point using the Student’s *t*-test in R (“*t*.test”) to identify significant differences in relative strain abundance.

#### Analysis of Probability of Recovering Multiple Subtypes From a Mixed Sample

Experimental frequencies from each subtype rank were averaged to establish the mean probability of successfully selecting each subtype based on picking one colony from a mixed plate post recovery using both enrichment and non-enrichment methods. These probabilities were then combined to compare posterior binomial probabilities of selecting at least one of each subtype from a mixed plate after each recovery method as a function of the total number of colonies sampled.

## Results

### Analysis of Subtype Prevalence in *Campylobacter* Databases

**FIGURE 1:**
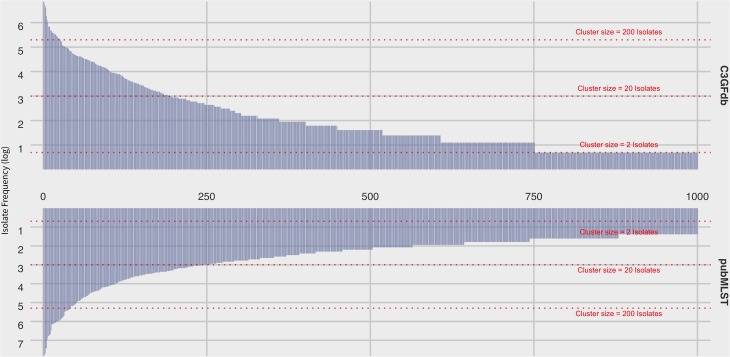
Frequency distribution of subtype clusters from the Canadian *Campylobacter* Comparative Genomic Fingerprinting database (C3GFdb) and the *C. jejuni* Multilocus Sequence Type database (pubMLST). Each bar along the horizontal axis represents a unique C3GFdb Comparative Genomic Fingerprint subtype or MLST Sequence Type. The top 1000 subtypes, in descending order by overall frequency, are shown. Visual guides are provided to indicate various cluster size ranges. A log scale is used.

**FIGURE 2:**
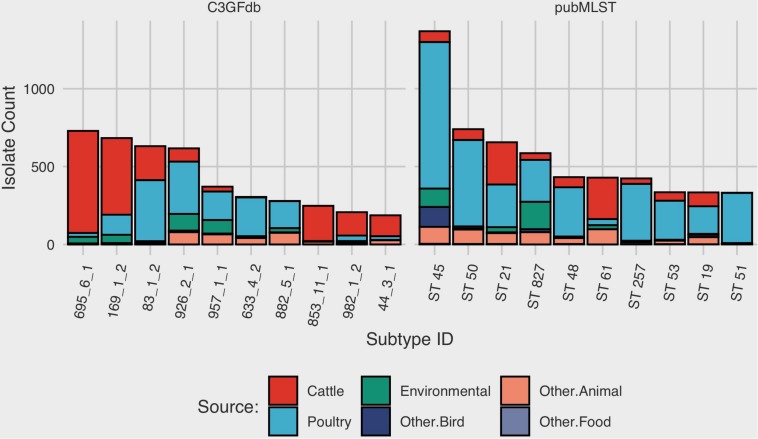
Source-distribution bias among common subtypes in the Canadian *Campylobacter* Comparative Genomic Fingerprinting and the *C. jejuni* Multilocus Sequence Type databases. Each bar along the horizontal axis represents a unique Comparative Genomic Fingerprint subtype or MLST Sequence Type. The top 10 subtypes, in descending order by overall frequency, are shown.

At the time of writing (Oct. 2019), the C3GFdb contained information on a pan-Canadian collection of 23,142 isolates comprising 5,037 distinct CGF subtypes from a wide range of sources including animal, human, and environmental origin. Similarly, the *Campylobacter* pubMLST database contained information on 72,806 *Campylobacter* isolates with 9,778 distinct Sequence Types (STs), also from a diverse range of international sources. Examination of the distribution of subtype frequencies observed within these databases revealed that a small number of subtypes have contributed disproportionately toward the overall number of entries in each database (Figure 1). For example, the ten most common subtypes accounted for 22% (*n* = 5099/23142) of the total isolates in the C3GFdb, with 73% (*n* = 3685/5037) of CGF subtypes observed in only single isolates (“singletons”), and over 92% of subtypes (*n* = 4648/5037) representing small clusters of five or fewer isolates. A similar trend was observed in the pubMLST database, whereby the ten most common subtypes represented nearly 27% (*n* = 19542/72806) of the total isolates in the collection. Meanwhile, nearly 66% of subtypes in this database (*n* = 6445/9778) had only been observed as singletons, and over 91% of subtypes (*n* = 8952/9778) representing small clusters of five or fewer isolates. To explore whether the subtype distribution observed in both the C3GFdb and pubMLST was associated with single-source sampling, we assessed the source distributions of the ten most prevalent subtypes in each database, in order to identify any subtypes with very narrow source range. No evidence of source restriction was observed among prevalent subtypes, as each one represented isolates derived from human and multiple non-human sources (Figure 2).

### Microbiological Competitive Recovery Experiments

**FIGURE 3:**
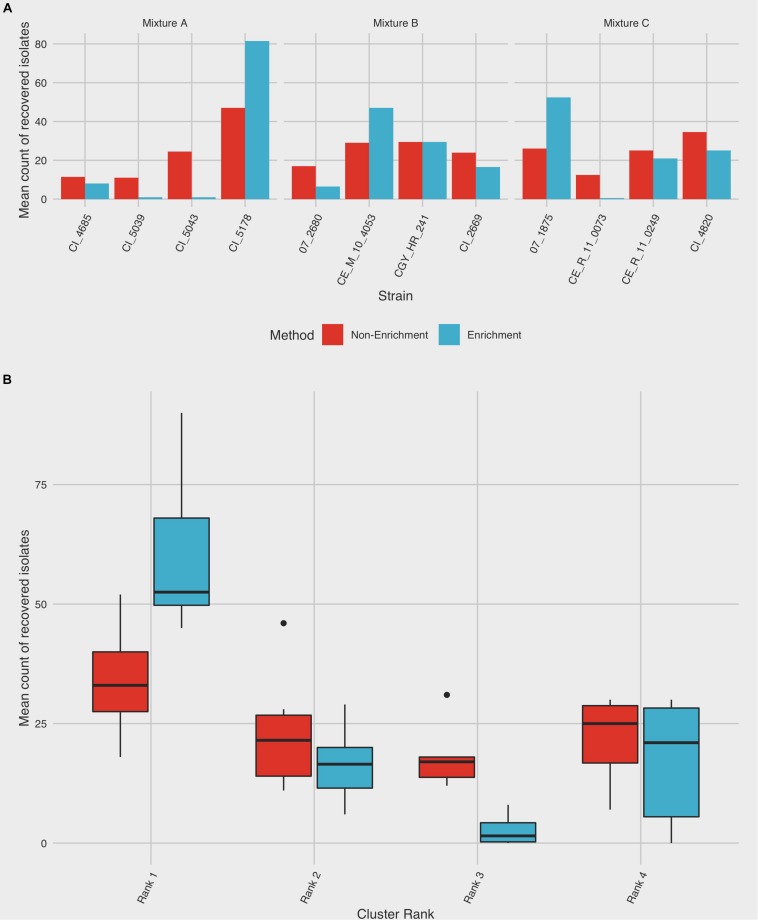
Summary of *C. jejuni* subtype recovery using enrichment and non-enrichment. Colonies were recovered from three different mixtures comprising strains from four distinct CGF subtypes using enrichment and non-enrichment methods and subjected to subtype-specific enumeration to assess relative rates of recovery. Each mixture was tested in duplicate; the frequency of isolation is based on 100 colonies selected per replicate. **(A)** The average frequency of recovery between the two trials is shown for each strain. **(B)** Frequency of recovery by subtype ranking, which was derived from prevalence in the Canadian *Campylobacter* Comparative Genomic Fingerprinting database (C3GFdb). Boxes represent the first and third quartiles and are split by a line representing the median; whiskers extend to 1.5x the interquartile range of the data. Outliers outside of this range are denoted individually as points.

In each of the pooled mixtures, the mean number of isolates from a single dominant subtype increased following an enrichment step, compared to isolates from the remaining three subtypes, which collectively decreased in relative frequency of recovery (Table 1 and Figure 3A). Chi-square goodness of fit test showed a significant deviation from the null hypothesis that equal or similar proportions of isolates from each of the 12 strains tested would be recovered after both non-enrichment (*χ*^2^ = 99.23, df = 11, *p* < 0.0001) and enrichment (*χ*^2^ = 572.37, df = 11, *p* < 0.0001) isolation procedures. Analysis of variance indicated that a significant difference (*p* < 0.0001) existed between the total number of recovered isolates for each subtype within each of mixtures A-C, and an interaction was observed between subtype and isolation method (*p* < 0.0001), suggesting that the mean frequency of recovered subtypes differed based on the isolation method used (Table 1 and Figure 3A). Follow-up pairwise analyses indicated that the recovery of strains CI_5178 (mixture A) and 07_1875 (mixture C) differed significantly based on isolation method used (*p* = 0.0012 and *p* = 0.0265, respectively). The frequency of strain CI_5043 (mixture B) did not differ significantly at a 95% significance level (*p* = 0.0762), but this result still suggested that isolation method may affect the recovery frequency of this strain.

When ranked as a function of subtype cluster frequency from the C3GFdb, the mean number of isolates from subtypes designated “Rank 1” increased from 34.00 (95% CI: 21.5–46.5) following non-enrichment recovery to 60.3 (95% CI: 42.0–78.7) following enrichment (Figure 3B). A significant effect for the recovery of isolates was found for “Subtype Rank” (*p* < 0.0001), and follow-up analyses revealed that subtypes selected from “Rank 1” in each experimental mixture were recovered in higher amounts than any of the three remaining subtypes (*p* < 0.0001) while no differences were found between the recovery of isolates from subtypes ranked two to four. Furthermore, when including the effect of “Isolation Method” in the model (i.e. enrichment vs. non-enrichment), an interaction with “Subtype Rank” was found (*p* = 0.0002), suggesting that the recovery of isolates based on subtype ranking significantly differed based on the isolation method used. Post hoc follow-up indicated that only the recovery of subtypes from the largest subtype clusters (“Rank 1”) differed significantly as a function of isolation method (*p* = 0.0048), suggesting that isolates from the largest clusters were recovered at a significantly higher frequency when an enrichment step was used in the isolation procedure (Figure 3B).

### Quantification of Strain-Specific DNA in Mixed Culture Using Digital Droplet PCR

**FIGURE 4:**
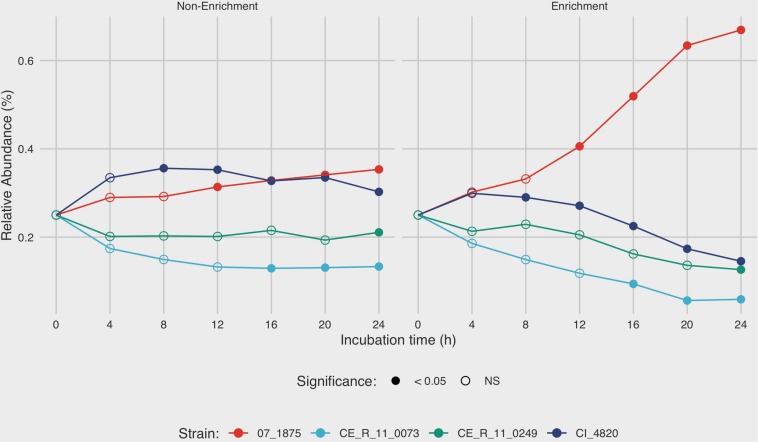
Analysis of growth dynamics of a mixed culture under enrichment and non-enrichment conditions via digital droplet PCR (ddPCR). Strain mixture “C” was grown in enrichment and non-enrichment broth with DNA extracted at 4-h intervals over a 24-h incubation period. For each time point, strain specific-DNA was quantified using ddPCR to estimate mean relative abundance of each strain in the mixture. After 4 h in non-enrichment recovery, moderate differences were observed between the growth rates of the four strains, with final relative abundances ranging from 13.3 to 35.3% after 24 h. The mean of three replicates is shown. Filled circles denote time points in which there is significant difference (*p* < 0.05) in relative abundance between enrichment and non-enrichment recovery methods.

After 4 h in non-enrichment recovery, moderate differences were observed between the growth rates of the four strains in mixture C, with final relative abundances ranging from 13.3 to 35.3% after 24 h (Figure 4). Under enrichment conditions in BB, the differences in relative abundances of the four strains were much more pronounced. After 8 h of incubation in BB, the relative amounts of strain 07_1875 increased disproportionately to the other strains in mixture C. At the final measurement of 24 h, *C. jejuni* strain 07_1875 comprised almost 67% of the total DNA in the mixture. This is in contrast to the other three strains in the mixture, with each experiencing a decline in relative abundance after the 8-h incubation measurement. Several significant differences in measurements were observed when comparing the relative abundances for each strain between enrichment and non-enrichment recovery methods. In particular, the growth of strain 07_1875 significantly increased under enrichment starting at the 12-h mark when compared to the growth of the same strain under non-enrichment. By contrast, strains CI_4820, CE_R_11_0073, and CE_R_11_0249 demonstrated significantly lower relative abundances in the enrichment recovery compared to non-enrichment, beginning at the eight, 16 and 24-h time-points, respectively (Figure 4).

### Predicted Probability of Recovering Multiple Subtypes From Mixed Samples

**FIGURE 5:**
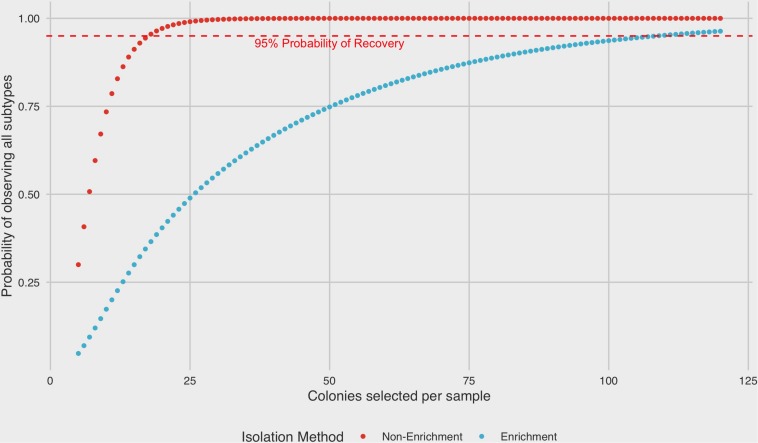
Binomial probability distribution of selecting all subtypes present in a mixed sample as a function of isolation protocol. A minimum of 18 colonies would need to be selected in order to recover at least one colony from each subtype in a four-strain mixed sample by non-enrichment isolation (95% probability level). In contrast, 109 colonies would be required to recover at least one colony from each subtype by enrichment.

A binomial probability distribution was constructed based on the results of ranked recoveries to assess the posterior probability of recovering colonies from all four subtypes present in a sample mixture by either enrichment or non-enrichment isolation methods. Under non-enrichment isolation, we found that a selection of a minimum of 18 isolates was required to recover at least one colony from each of four subtypes present with 95% probability. By contrast, when performing isolation following a pre-enrichment step, the selection of 109 colonies was required to recover at least one colony from each subtype with 95% probability (Figure 5).

## Discussion

Studies assessing the prevalence of *C. jejuni* subtypes from animal, food and environmental sources have shown that a select number of subtypes are commonly found; globally, the most consistent of these lineages have been characterized as belonging to MLST sequence types ST-45 and ST-21 (Dingle et al., 2002; Kwan et al., 2008; Sheppard et al., 2009; Müllner et al., 2010; Gripp et al., 2011). These subtypes are often characterized by their diverse host association and high prevalence worldwide and have thus been termed “generalists” due to their pervasive nature across ecologic niches and apparent adaptability to diverse conditions (Sheppard et al., 2011, 2013). Although high frequency of isolation of certain subtypes may be truly reflective of their prevalence throughout the sampled environments, it is also possible that augmented rates of recovery observed for some subtypes may at least be partly due to the selective advantage conferred by the isolation method used. Anecdotally, we have previously observed limited genotypic diversity among isolates recovered from water samples potentially impacted by agricultural activities and thus expected to reflect these inputs. To experimentally address the possibility of subtype-specific recovery bias, we empirically assessed the effects of a commonly used enrichment-based culture method on the recovery of *C. jejuni* subtypes *in-vitro* from multi-strain samples using select isolates from Canadian surveillance (i.e. C3GFdb).

Recovery experiments were performed under controlled laboratory conditions using sterile Bolton’s enrichment broth (BB) spiked with normalized quantities of broth culture from four *C. jejuni* strains of known subtype. While previous studies have documented differences in genotype diversity when comparing isolation procedures (Devane et al., 2013; Ugarte-Ruiz et al., 2013), our experimental design included several features selected to avoid confounding factors related to the analysis of field-collected samples. These included: (a) the use of mixtures composed of known *Campylobacter* subtypes, which avoided complications related with the analysis of samples comprising unknown subtype composition; (b) the use of controlled culture conditions, which avoided issues related to different sample matrices or background organisms present in samples collected from the field; (c) the use of BB, which was selected for this study because it was used in the original culturing of isolates used in our experiments; BB is one of the most widely used enrichment mediums for the culturing of *Campylobacter* species and it is recommended for use by the International Standard Organization (International Organization for Standardization, 2006) and the U.S. Food and Drug Administration (Hunt et al., 1998).

Under the null hypothesis that no recovery bias exists for either the enrichment or non-enrichment isolation method, the recovery of similar proportions of each of the four subtypes within each cohort was expected. While we anticipated that recovery using enrichment may bias the growth of particular *C. jejuni* strains, surprisingly, we observed significant variation in the recovery of the four strains present after non-enrichment recovery as well. This variation was likely in part due to limitations in the experimental set-up (e.g. normalization of bacterial inoculum using OD_600_, inherent differences in growth rate between the individual strains, stochastic variation, etc.). However, similar co-culturing experiments have shown biased growth competition between strains independent of growth rate or initial inoculum, illustrating complex inter-strain dynamics (Mellefont et al., 2008; Zilelidou et al., 2015). Importantly, the results from the non-enrichment isolation experiments in this study served as a useful baseline for assessing results from the enrichment-based recovery experiments. In all enrichment recovery experiments conducted, we observed a statistically significant deviation from the null hypothesis, and the extent of this bias was much more pronounced compared to that observed when using the non-enrichment isolation procedure. This suggests that employing an enrichment step may preferentially enhance the recovery of particular subtypes either by directly favoring their growth or by hampering the growth of others.

The experimental design of the recovery experiments only included measurement by spectrophotometer (OD_600_) to provide estimates for normalizing strain concentrations prior to pooling the starting inoculum. To deliver more quantifiable evidence for the differences in strain growth dynamics under enrichment and non-enrichment, we designed an additional experiment for one of the strain mixtures using a digital droplet PCR system (ddPCR). Using a similar experimental design, including normalization of broth inoculum via OD_600_, we again noticed variation in copy number estimates of each strain at *t* = 0; these initial estimates ranged from 18-31% of the total abundance of the original inoculum. These results indicated that, as expected, OD_600_ alone provided an insufficient means of normalizing concentrations of the broth cultures. However, one of the advantages of the ddPCR approach was the ability to control for these discrepancies in the analysis stage, thereby providing estimates of relative strain growth unaffected by the differences in the original inoculum. After applying analytical input controls, we still observed trends in the growth of the four strains in mixture similar to that seen in the culturing experiments, suggesting that overall, the results from the original microbiological culturing methods were likely sound, although the exact estimates should be interpreted with caution. As observed in the original experiments, we observed significant differences in the growth profiles of the four strains measured using ddPCR that were exacerbated by the use of enrichment media. The clinical strain 07_1875 performed significantly better under enrichment conditions compared to the remaining strains, whose growth by comparison seemed to suffer under enrichment. Interestingly, at *t* = 24 h, the order of relative abundance for each strain remained constant under both recovery methods, though magnitudes differed significantly, suggesting that each of these four strains may have inherently different growth rates under the laboratory conditions used.

To test the hypothesis that high subtype frequency in surveillance data may at least in part reflect selection bias under laboratory conditions, overall recovery frequencies from all microbiological recovery experiments were compared by assigning the subtypes in each mixture a rank in descending order from “1” to “4” corresponding to the relative frequency of each subtype in the C3GFdb. We observed a substantial difference in recovery frequency when comparing the results from non-enrichment isolation versus enrichment noting that the “Rank 1” subtypes increased in frequency from 40.5% of recovered isolates using non-enrichment recovery to 62.4% under enrichment. This is in contrast to the frequency of recovery observed for the remaining subtypes (Ranks 2, 3 and 4), which collectively decreased from 59.5% of the population recovered by non-enrichment isolation to 37.6% of isolates by enrichment. These results are consistent with studies by Williams et al. (2012) and Ugarte-Ruiz et al. (2013) in which they assessed the genotype diversity of *C. jejuni* isolated from poultry samples using a variety of isolation protocols and found method-specific differences in the genotypes and numbers of isolates observed. They concluded that enrichment methods, in particular, limit the “genotypic richness” of the population recovered (Williams et al., 2012; Ugarte-Ruiz et al., 2013). In contrast to using natural samples for experimentation, our controlled experimental set-up allowed us to examine the nature of these findings more directly owing to the removal of potential confounders when dealing with recovery of isolates from samples of unknown strain composition.

A primary concern was that the physiology of isolates sampled from different ecologies may impact their performance in the laboratory, e.g. isolates recovered from river water may not perform well at laboratory temperatures that more closely reflect those of avian or human intestinal tracts. Thus, in designing each of the experimental strain mixtures, we attempted to mitigate effects related to the original source of sampling by creating mixtures that contained isolates sampled from environmental, food-animal, and human clinical sources. As such, when selecting isolates from highly prevalent subtypes, we attempted to ensure that mixtures represented isolates from subtypes of both single and mixed origins. Our results did not appear to indicate systematic bias related to the original sampling source of isolates used in our experiments, though a separate analysis would be required to fully assess this.

While it may not be surprising that certain subtypes could be better adapted to laboratory conditions, leading to higher isolation frequencies in microbiological surveillance, the more significant implication is the much-reduced chance of recovery of other subtypes as a result of being out-competed under standard isolation conditions. The international standard ISO 10272:2006 recommends up to 5 well-formed colonies be selected for subtyping analysis (International Organization for Standardization, 2006; Habib et al., 2011). We have recently shown that specialized isolation methods resulted in a greater than 2-fold increase in culture-positive diarrheic stools in samples submitted for diagnostic testing in Southwestern Alberta when compared to conventional diagnostic methods used across Canada (Inglis et al., 2019). Anecdotally, many laboratories operate under a resource-constrained environment that may preclude implementing this recommendation. Based on the frequencies of each subtype rank from our experimental results, we found that selecting only five colonies resulted in a 70% chance of missing at least one of the subtypes present in a mixed-strain sample when performing recovery without BB enrichment, and this rose to 95% when using enrichment with BB. Our model suggests that in order to increase the likelihood of identifying less common subtypes recovery protocols should include: (1) employing culturing methods that do not contain an enrichment step or performing parallel enrichment- and a non-enrichment-based methodologies, and (2) increasing the number of colonies selected for subtyping. However, the results derived here should be interpreted with caution, given practical limitations surrounding laboratory procedures in these experiments and the need for more comprehensive examination of the kind of dynamics described here using a wider selection of strains and additional culture conditions, which should be performed to determine the extent of potential bias in surveillance data.

## Conclusion

Findings from the current study demonstrate that culture methods for the isolation of *C. jejuni* can bias the recovery frequency of isolates from mixed strain samples by obscuring the presence of less common subtypes while over-representing others, an effect that can be exacerbated by the use of enrichment-based protocols. Importantly, this bias may compromise our ability to identify subtypes of high public health significance but low adaptation to laboratory conditions. Although this is not likely to have a direct impact on patient treatment given the lack of routine *C. jejuni* subtyping to inform diagnosis or epidemiological follow-up in most jurisdictions, microbiological surveillance can play an essential role in the development of mitigation strategies to reduce the incidence of campylobacteriosis through the identification of potential sources of exposure, transmission vehicles, and reservoirs. Our findings underscore the need for further research to help minimize bias from findings based on culture-based microbiological surveillance.

## Data Availability Statement

The raw data supporting the conclusions of this article will be made available by the authors, without undue reservation, to any qualified researcher.

## Author Contributions

BH participated in all aspects of laboratory and downstream analyses and drafted the manuscript. SM participated in laboratory work and drafting the manuscript. CC, JT, GI, VG, and ET contributed to study design, funding and/or writing the manuscript.

## Conflict of Interest

The authors declare that the research was conducted in the absence of any commercial or financial relationships that could be construed as a potential conflict of interest.

## Footnotes

1 https://primer3.org/
